# Oxidative Stress Induced in Sunflower Seedling Roots by Aqueous Dry Olive-Mill Residues

**DOI:** 10.1371/journal.pone.0046137

**Published:** 2012-09-26

**Authors:** Inmaculada Garrido, Mercedes García-Sánchez, Ilda Casimiro, Pedro Joaquin Casero, Inmaculada García-Romera, Juan Antonio Ocampo, Francisco Espinosa

**Affiliations:** 1 Departamento Biología Vegetal, Ecología y Ciencias de la Tierra, Universidad de Extremadura, Badajoz, Spain; 2 Departamento Microorganismos Rizosféricos Promotores del Crecimiento Vegetal, Estación Experimental Zaidín, CSIC, Granada, Spain; 3 Departamento Anatomía, Biología Celular y Zoología, Universidad de Extremadura, Badajoz, Spain; National Taiwan University, Taiwan

## Abstract

The contamination of soils with dry olive-mill residue can represent a serious problem as being an environmental stressor in plants. It has been demonstrated that inoculation of aqueous extract of olive oil-mill residue (ADOR) with saprobe fungi removes some phenolic compounds. In this paper we studied the effect of ADOR uninoculated or inoculated with saprobe fungi in sunflower seedling roots. The germination and root growth, O_2_·^-^ generation, superoxide dismutase (SOD) and extracellular peroxidases (EC-POXs) activities, and the content of some metabolites involved in the tolerance of stress were tested. The roots germinated in ADOR uninoculated show a decrease in meristem size, resulting in a reduction of the root length and fresh weight, and in the number of layers forming the cortex, but did not alter the dry weight, protein and soluble amino acid content. ADOR caused the decreases in O_2_·^-^ generation and EC-POX′s activities and protein oxidation, but enhanced SOD activity, lipid peroxidation and proline content. Fluorescence imaging showed that ADOR induced O_2_·^-^ and H_2_O_2_ accumulation in the roots. The increase in SOD and the decrease in EC-POX′s activities might be involved in the enhancement of H_2_O_2_ content and lipid peroxidation. Control roots treated with ADOR for 10 min show an oxidative burst. Roots germinated in ADOR inoculated with saprobe fungi partially recovered normal levels of ROS, morphological characteristics and antioxidant activities. These results suggested that treatment with ADOR caused a phytotoxic effect during germination inducing an oxidative stress. The inoculation of ADOR with saprobe fungi limited the stress.

## Introduction

The oxidative burst is the controlled, rapid production of reactive oxygen species (ROS) occurring in response to stimulation of plant cells by biotic or abiotic stresses [1]. This response includes O_2_·^-^ and H_2_O_2_ release in the apoplast. Various enzyme systems have been proposed as responsible for generating ROS in the apoplast of plant cells and as playing crucial roles in several situations during plant growth and development [2],[3]. These include a trans-PM-NADPH-oxidase [4],[5], that catalyzes the one-electron reduction of extracellular molecular oxygen to O_2_·^-^, this being spontaneously or enzymatically (mediated by superoxide dismutase, SOD) dismutated to H_2_O_2_. It has been demonstrated that POX generates ROS in response to different stresses [6],[7]. In leaves and cell cultures of French bean, a POX has been cloned and characterized [8] which peroxides membrane fatty acids and then directly generates H_2_O_2_ in the apoplast. Lipid peroxidation and protein oxidation is often considered to be an invaluable marker of oxidative stress [9]. In plants, there is a close correlation between ROS and lipid peroxidation under environmental stress [10],[11] and protein oxidation [12], and under conditions of stress the proportion of carbonylated proteins increases [13]. Plants normally raise the levels of several components of their antioxidant system in response to stress. Proline accumulation has been described during oxidative stress [14],[15] in response to stress [16],[17], and several studies have attributed it an antioxidant character, including ROS scavenging activity [18],[19] and the potential to reduce lipid peroxidation in alga cells exposed to heavy metals [20]. Finally, an increase in the synthesis of phenolic compounds is another common response to environmental stress in plants [21], and flavonoids and phenylpropanoid glycosides (PPGs) could remove ROS [22],[23]. These compounds are powerful antioxidants either by direct scavenging of ROS or by stabilization and delocalization of the unpaired electron (chain-breaking function) [24].

Dry olive-mill residue (DOR) is of potential interest for use as a fertilizer because of its high organic matter content [25]. However it contains phytotoxins capable of inhibiting plant growth [26]. Most of these phytotoxins are phenolics [27], some of which have been shown to inhibit seed germination, seedling growth, root elongation, chlorophyll accumulation, and leaf expansion [28],[29]. Christensen et al. [30] showed that lignification in poplar xylem is correlated with peroxidases that are induced under stress, and treatment with phenolic compounds leads to a reduction in root growth that is associated with premature lignification, lipid peroxidation, and raised peroxidase and superoxide dismutase activities [29],[31],[32]. The contamination of soils with DOR can therefore represent a serious problem as being an environmental stress that induces defence reactions in plants, including an oxidative burst. A possible solution is to use biological methods such as bioremediation with saprobe fungi to remove the phenolic compounds from the DOR before its application. Sampedro et al. [33] and Aranda et al. [34],[35],[36] showed that the treatment of DOR with different saprobe fungi decreased the phytotoxicity because of the ability of these fungi to release extracellular enzymes involved in the removal of monomeric phenols.

In this paper we report the changes on germination and root growth of sunflower seedling. We evaluated if aqueous extract of olive oil mill residue (ADOR) application enhanced O_2_·^-^ generation, induced changes on SOD and EC-POX activities, and if some metabolites involved in the tolerance of ADOR stress in axenic seedling roots of sunflower germinated in ADOR with or without incubation with saprobe fungi.

## Materials and Methods

### Plant material and treatments

Sunflower (*Helianthus annuus* L.) seeds (Koipe, S.A., Sevilla, Spain) were surface sterilized, soaked, and germinated for 48 or 72 h in darkness at 27±1°C on filter paper moistened with sterilized distilled water [37] or ADOR non-incubated or incubated with saprobe fungi.

The dry olive-mill residue (DOR) was collected from an “orujo” manufacturer (Aceites Sierra Sur, Granada, Spain). The aqueous extract (100% ADOR) was obtained by orbital-shaking of the DOR with distilled water in the proportion 1∶2 (w/v) for 8 h, followed by filtering the suspension through several layers of cheesecloth [34]. The ADOR (100%) was used as growth medium for the saprobe fungi *Trametes versicolor* IJFM A136, *Coriolopsis rigida* (CECT 20449), *Pycnoporus cinnabarinus* IJFM A720 (CECT 20448) and *Penicillium chrysogenum* 10 (EEZ 10). The inoculum was produced by growing the fungus under orbital shaking at 125 rpm and 28°C on extract malt for 7 days. The mycelia was collected and homogenized with an Ultra turrax mixer. Each flask was inoculated with 5 mL of the inoculum. The fungi were grown in Erlenmeyer flask (250 mL) containing 70 mL of Medium Basal (MB) during 22 days at 28°C to produce ligninolytic and hydrolytic enzymes [38]. After 22 days the MB was supplemented with 70 mL of ADOR (100%) and was incubated for 15 days at 28°C. The culture liquid was separated from the mycelium by filtration through a disk of filter paper and the supernatant were used for measurement [34],[36]. The 50% ADOR is obtained by dilution of 100% ADOR in distilled water.

The different treatments used for germination were: distilled water (control), 50% ADOR non-incubated (ADOR), and 50% ADOR incubated with *P. cynnabarinus* (ADOR-Pc), *C. rigida* (ADOR-Cr), *P. chrysogenum-10* (ADOR-Pch), and *T. versicolor* (ADOR-Tv). For short-treatment with ADOR, control roots were incubated for 10 min, 1 h, 3 h or 24 h in 50% ADOR before the activities determination.

### Enzyme determinations

Assays were carried out on the intact seedling roots or on a crude extract of the roots. When intact roots were used, the roots of intact seedlings were carefully immersed directly in the corresponding reaction mixture, through a plastic mesh by which cotyledons covered by the unbroken seed coats were maintained outside the solution. For crude extracts, the roots were homogenized at 4°C in 50 mM phosphate buffer, 1 mM ethylenediaminetetraacetic acid (EDTA), 0.5 mM phenylmethylsulfonyl fluoride (PMSF), 1 mM β-mercaptoethanol, 1 g/L poly vinylpolypyrrolidone (PVPP), pH 6.0. The homogenate was filtered and centrifuged at 39000 *g* for 30 min at 4°C, the pellet was discarded, and the supernatant immediately used for the measurements. The protein content was determined by method of Bradford [39].

O_2_·^-^ generating activity of the roots was determined spectrophotometrically by measuring the oxidation of epinephrine to adrenochrome as A_480_ [40], with the reaction mixture containing 1 mM epinephrine in 25 mM acetate buffer, pH 5.0 (ε = 4.020 mM^−1^ cm^−1^).

Superoxide dismutase activity (SOD, EC 1.15.1.1) was determined as A_550_ in 50 mM phosphate buffer, 0.1 mM EDTA, 1 mM NaCN, 0.01 mM cyt c, and 1 mM xanthine, pH 6.0 [41]. A unit of SOD is defined as the amount of enzyme required to cause 50% inhibition of cytochrome *c* reduction.

Extracellular peroxidase activity (POD, EC 1.11.1.7), EC-POX, was measured at A_590_ (ε = 47.6 mM^−1^ cm^−1^) [42], with the reaction mixture containing 3.3 mM 3-dimethylaminobenzoic acid (DMAB) and 66.6 µM 3-methyl-2-benzothiazolinonhydrazon (MBTH) in 50 mM phosphate buffer pH 6.0. A unit of ECPOX is defined as the amount of enzyme required to cause the formation of 1 nmol DMAB-MBTH (indamine dye) per minute at 25°C, pH 6.0. The coniferyl alcohol (CA) EC-POX activity was recorded by measuring the decrease in absorbance as A_265_ of a reaction medium composed of 0.1 mM CA in 25 mM acetate buffer pH 5.0 (ε = 7.5 mM^−1^ cm^−1^). A unit of CA-ECPOX is defined as the amount of enzyme required to cause the oxidation of 1 nmol CA per minute at 25°C, pH 5.0.

### Biochemical analysis

Total flavonoid content was measured colorimetrically [43] with minor modifications. Aliquots (200 µL) of sample [44] were mixed with 800 µL H_2_0 and 60 µL 5% NaNO_2_. After 5 min, 60 µL of 10% AlCl_3_ solution and 400 µL 1 M NaOH were added, the reaction solution was well mixed and left to stand for 15 min, the absorbance was determined at 415 nm, and the total flavonoid content was calculated using the standard rutin curve and expressed as µg of rutin mg^−1^ DW.

PPGs were determined by a colorimetric method based on estimating an *o*-dihydroxycinnamic derivative using the Arnow reagent as follows: 150 µL of sample [44] was mixed with 300 µL 0.5 M HCl, 300 µL Arnow reagent, 300 µL 2 M NaOH, and 450 µL H_2_O; after 10 min, the A_525_ was measured and the concentration was calculated on the basis of the standard curve of 3,4-dihydroxyphenylalanine, and expressed as µg verbascoside mg^−1^ DW [45].

Ferric reducing antioxidant power (FRAP) determination was performed at A_593_ as described in Rios et al. [44]. Calibration was against a standard curve using freshly prepared ammonium ferrous sulfate [46], and the concentration was expressed as µg ferrous sulfate mg^−1^ DW.

Free radical scavenging activity was determined at A_517_ using the 2,2-diphenyl-1-picrylhydrazyl (DPPH) test [47], and the percentage of free-radical scavenging effect calculated.

Lipid peroxidation was determined by measuring malondialdehyde (MDA) formation using the thiobarbituric acid (TBA) method described by Madhava Rao and Sresty [48]; the MDA concentration was calculated by using an ε = 155 mM^−1^ cm^−1^, and expressed as µmol MDA mg^−1^ DW.

The carbonyl content was determined by the 2,4-dinitrophenylhydrazine technique [49]; and the carbonyl content was obtained by measuring the absorbance at A_370_ (ε = 22000 M^−1^ cm^−1^).

Proline concentration was estimated by the method of Irigoyen et al. [50] optimized: 1 g of roots was homogenized with 5 mL of absolute ethanol and washed twice with 2.5 mL 70% ethanol to a final volume of extraction of 10 mL; the homogenate was centrifuged for 10 min at 3600 *g*; and proline content was obtained by measuring at A_515_, calculating the concentration on the basis of the standard curve of proline and expressing it as µg proline mg^−1^ DW.

For the amino acid and soluble-protein determination, 0.5 g of roots was homogenized with 5 mL of cold phosphate buffer (50 mM, pH 7.0) and centrifuged at 12000 *g* for 15 min; the supernatant was used to determine total amino acids by the ninhydrin method as described by Ruiz et al. [51]; total free amino acids were expressed as mg glycine g^−1^ FW.

### Root tip sections

Root tips for sequential sectioning were fixed for 3 h at room temperature in 4% glutaraldehyde, 4% formaldehyde, and 50 mM phosphate buffer, pH 7.2. Serial ethanol dehydration was then performed (30, 50, 70, 90, and 100% [twice]) for 1 h at each step. Samples were embedded in Spurr's resin. Sequential serial sections of 5 µm were cut, dried on glass slides, and stained in 0.05% aqueous toluidine blue-O solution at 60°C for 30 s (adapted from Burgos et al. [52]).

### Imaging of reactive oxygen species

Root segments of approximately 20 mm were cut from the apex and incubated for 30 min at 37°C in darkness, with 30 µM 2′,7′-dichlorofluorescein diacetate (DCF-DA, peroxide accumulation) or 15 µM dihydroethidium (DHE, superoxide accumulation) in 10 mM Tris-HCl (pH 7.4), and washed twice for 10 min each in the same buffer [53]. After washing, the roots were observed under fluorescence microscopy (Axioplan-Zeiss microscope) to visualize the whole root. As negative control, roots were pre-incubated before adding the probes in darkness for 60 min at 25°C, with 1 mM ascorbate (peroxide scavenger) or 1 mM tetramethyl piperidinooxy (TMP, superoxide scavenger).

### Statistical analysis

The data presented are the means±SD of at least 10 replicates obtained from three independent experiments. The data obtained were statistically analyzed by the Mann-Whitney U test.

## Results

The stress caused by ADOR reduces the germination rate and root lengths, with intermediate values for the other treatments (Table 1). Thus, while the control presented a germination rate of 89.91%, treatment with 50% ADOR reduces this percentage to 20.65%. Root lengths were reduced by between 56.6% and 32.5% for ADOR and ADOR-Tv, respectively, giving intermediate values for the other treatments. A similar trend was observed for FW of the roots, but not for DW. In the protein content, there were no significant differences between treatments, although there was an apparent tendency of increased protein content in roots from the ADOR treatments. For the soluble amino acid content, ADOR and ADOR-Tv levels were lower than the control. The amount of proline increased with increasing ADOR treatments, this increase was lower in ADOR incubated with saprobe fungi. All ADOR treatments increased in flavonoid levels but decreased in PPGs levels, except for ADOR-Cr. Total antioxidant capacity showed a sharp decline (≈36%) in germination rate induced by ADOR.

**Table 1:** Effects of treatments of 50% ADOR (incubated or not with saprobe fungi) on the germination, root length, root fresh and dry weight, total protein, soluble aminoacid and proline content, total flavonoids and PPGs and antioxidant capacity (FRAP and DPPH test) of sunflower seedling roots germinated during 48 h or 72 h (^1^).

Treatment	Control	ADOR	ADOR-Pc	ADOR-Cr	ADOR-Pch	ADOR-Tv
Germination (%)	89.91±3.34	20.65±7.37^a^	72.48±6.84	78.43±4.50	69.94±5.64^a^	84.08±6.15
Germination (%)1	92.06±3.41	48.06±6.10^a^	84.11±5.52	86.00±2.91	87.34±3.70	89.81±5.20
Root length (cm)	2.03±0.39	0.88±0.53^a^	1.17±0.27^a^	1.20±0.52^a^	1.31±0.29^a^	1.37±0.47^a^
Fresh weight (mg)	20.66±3.10	8.57±0.91^a^	11.54±1.30^a^	10.93±0.42^a^	10.62±0.71^a^	11.18±0.60^a^
Dry weight (mg)	1.39±0.09	1.32±0.15	1.36±0.15	1.29±0.05	1.27±0.14	1.32±0.09
Protein content (µg/mg DW)	43.27±6.40	43.93±5.00	44.23±6.80	45.07±2.50	44.52±8.60	43.60±7.20
Soluble amino acid (µg glycine/mg DW)	212.40±39.70	173.96±49.60^a^	222.45±63.20	236.65±61.50	241.34±60.40	187.74±60.00
Proline content (ng/mg DW)	3.36±0.20	15.43±1.20^a^	10.04±2.90^b^	11.55±0.90^b^	10.73±2.90^b^	10.01±0.80^b^
		10.69±2.70^b2^				
Total flavonoids (µg rutin/mg DW)	21.17±4.70	25.68±3.80	22.35±3.60	26.05±6.40	24.16±1.80	23.64±1.90
Total PPGs (µg alanine/mg DW)	27.69±6.40	24.39±4.50	22.65±3.60	26.43±3.80	23.69±3.50	25.45±4.60
FRAP (µmols SO4Fe/mg DW)	0.306±0.040	0.198±0.007^a^	0.219±0.007^a^	0.241±0.010^b^	0.251±0.007^b^	0.263±0.020^b^
DPPH (%/mg DW)	24.61±1.81	9.53±2.10^a^	14.32±1.70^b^	15.09±1.45^b^	14.78±2.13^b^	17.51±2.07^b^

50% ADOR were used, except (**^2^**) 10% ADOR.

Mean±SD values (N = 5) followed by the same letter (^a-c^) are not significantly different according to Mann-Whitney U test (P≤0.05), between control and treatments.

**Figure 1:**
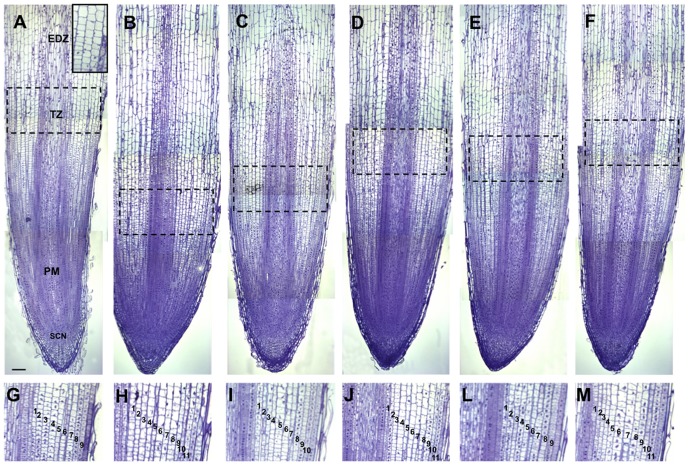
Apical meristems of *Helianthus annuus* roots. (A–F) Median longitudinal sections of root apices: (A) control, (B) ADOR, (C) ADOR-Pc, (D) ADOR-Cr, (E) ADOR-Pch, (F) ADOR-Tv; EDZ, elongation-differentiation zone; TZ, transition zone; PM, proximal meristem; SCN, stem cell niche. The insert shows a detail of elongating cells exiting from the meristem at the TZ. Scale bar: 100 µm. (G–M) Detail of cortex in (G) control and (H–M) treated plants showing the number of cortical cell layers: (H) ADOR, (I) ADOR-Pc, (J) ADOR-Cr, (L) ADOR-Pch, (M) ADOR-Tv.

Median longitudinal sections of root apices of sunflower showed an open organization (Figure 1). The limit between the stele and cap columella is poorly defined, as occurs in closed meristems. The columella arises from short files of cells from which repeated transversal divisions originate (stem cell niche, SCN). Files of large diameter cells that give rise to central metaxylem vessels are visible on the axis of the root apical meristem (RAM). Root cells first undergo repeated rounds of division in the root's proximal meristem, and then undergo rapid cell expansion in the elongation–differentiation zone (EDZ; Figure 1A). Cell differentiation is initiated in the transition zone (TZ), encompassing the boundaries between dividing and expanding cells in the different files [54],[55],[56]. According to the root transition zone concept, root cells leaving the apical meristem need to attain a transitional state in order to perform rapid cell elongation.

Although the organization of the root apex was the same in control and treated plants, they differed in RAM size. The results (Figure 1A–F) reveal a decrease in meristem size in treated plants. The TZ in control and ADOR, ADOR-Pc, ADOR-Cr, ADOR-Pch, and ADOR-Tv treated roots is located at approximately 1600, 890, 1100, 1350, 1220, and 1400 µm from the SCN, respectively. The treated roots, except for ADOR-Pch, also had more cortical cell layers (Figure 1G–M) due to extra longitudinal divisions occurring in the outer cortex.

**Figure 2:**
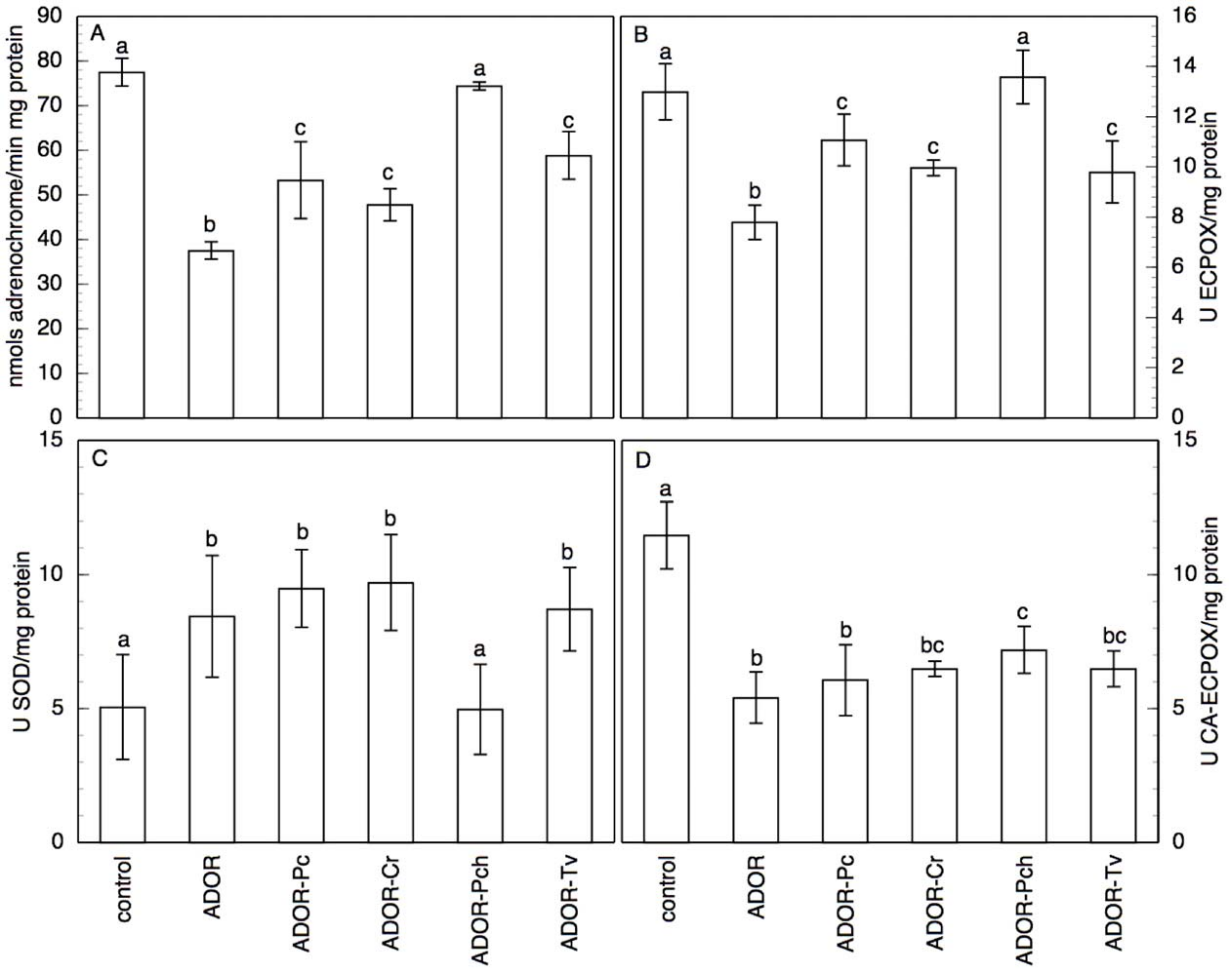
Oxidant and antioxidant activities in intact sunflower seedling roots. (A) O_2_·^-^ generation, (B) SOD, (C) DMAB-MBTH EC-POX, and (D) CA EC-POX activities for intact seedlings roots germinated and grown for 48 h in the different ADOR treatments. Data are means±SD of 5 independent experiments, each performed in triplicate. The means followed by the same letter are not significantly different according to Mann-Whitney U test (P≤0.05).

**Figure 3:**
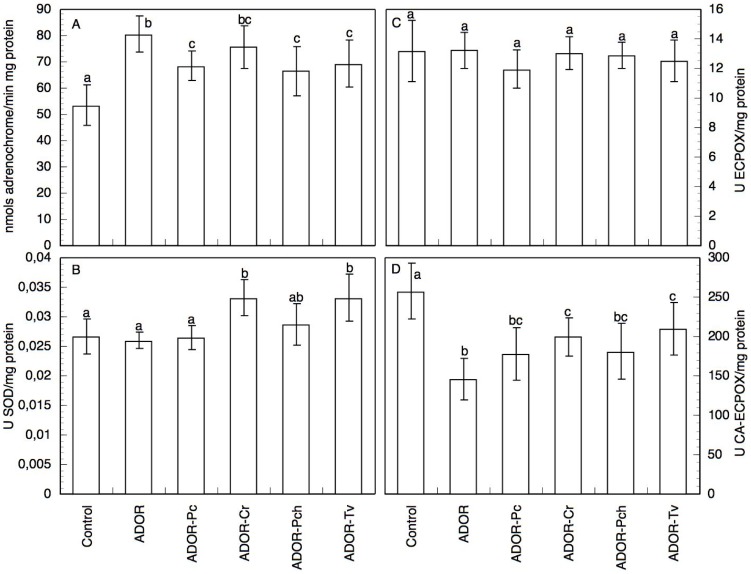
Oxidant and antioxidant activities in homogenates of sunflower seedling roots. (A) O_2_·^-^ generation, (B) SOD, (C) DMAB-MBTH EC-POX, and (D) CA EC-POX activities for homogenates of sunflower seedling roots germinated and grown for 48 h in the different ADOR treatments. Data are means±SD of 5 independent experiments, each performed in triplicate. The means followed by the same letter are not significantly different according to Mann-Whitney U test (P≤0.05).

The apoplast O_2_·^-^ generation increased 1.2-fold in response to 3 h ADOR treatment in control roots (Table 2), which is indicative of oxidative burst. After 48 h germination in ADOR, the roots showed a marked reduction in O_2_·^-^ generation, which is less significant with ADOR-Pc, ADOR-Cr, and ADOR-Tv, and that is recovered to values close to those of the control with ADOR-Pch (Figure 2A). This same activity showed an entirely different pattern of behaviour when determined in the homogenates (Figure 3A) of roots from the different ADOR treatments. There was a marked increase in O_2_·^-^ production in all the cases, but especially in the homogenate from the ADOR and ADOR-Cr germinated roots. The rest of the treatments presented higher values than control, but somewhat lower than the above two cases, evidence of a milder oxidative burst.

**Table 2:** Effects of treatment at different times with 50% ADOR on superoxide generation, SOD, ECPOX and CA-ECPOX activities in intact seedling roots.

Treatment	nmols adrenochrome/min mg protein	USOD/mg protein	U ECPOX/mg protein	U CA-ECPOX/mg protein
Control	77.427±3.104	5.663±0.136	12.341±1.109	11.279±1.349
C+10 min ADOR	85.309±2.517^a^	9.892±1.581^a^	14.306±1.240^a^	7.095±0.947^a^
C+3 h ADOR	92.959±4.055^b^	8.808±1.497^a^	14.697±1.467^a^	5.639±1.205^b^
ADOR	36.198±0.183^c^	7.649±0.911^b^	6.817±0.643^b^	4.985±0.956^b^

Mean±SD values (N = 5) followed by the same letter (^a,b^) are not significantly different according to Mann-Whitney U test (P≤0.05), between control and treatments.

The SOD activity increases by treatment for 10 min with ADOR (Table 2). In the ADOR roots (48 h germination), the increase was about of 1.6 over the control values (Figure 2B), and by a factor greater than 1.75 in the cases of ADOR-Pc and ADOR-Cr. The determination of this activity in homogenates (Figure 3B) gave values similar to the controls for ADOR and ADOR-Pc. The EC-POX activities presented the opposite trend to that observed for SOD. Thus, although the EC-POX activity increased by short-time treatment with ADOR, this activity was greatly reduced by germination in ADOR (Figure 2C). Roots germinated in ADOR treated with saprobe fungi showed higher activities than those in untreated ADOR. With respect to the CA-POX activity, both the roots treated for 10 min, 3 h and those germinated in ADOR (Table 2, Figure 2D) showed sharp declines. ADOR-Pc presented values similar to those obtained with untreated ADOR. In the homogenate (Figure 3C–D), while the CA-POX activity showed a similar trend to that obtained with the intact roots, the EC-POX activity showed a different behaviour with values similar to the control.

**Figure 4:**
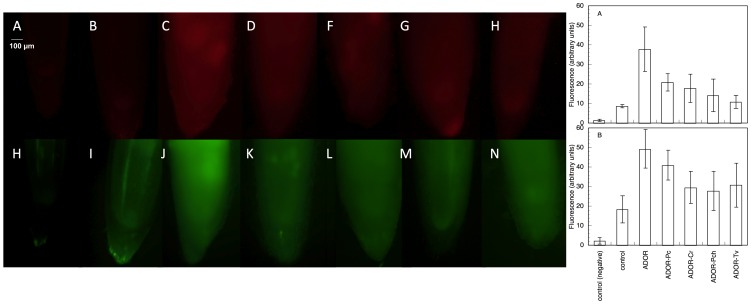
Imaging of ROS in sunflower roots. (A–G) Imaging of O_2_·^-^-dependent DHE and (H–N) peroxide-dependent DCF-DA fluorescence accumulation in sunflower roots germinated and grown for 48 h in the different ADOR treatments. (A,H) Negative control, (B,I) control, (C,J) ADOR, (D,K) ADOR-Pc, (E,L) ADOR-Cr, (F,M) ADOR-Pch and (G,N) ADOR-Tv. Scale bar: 100 µm. The figures show fluorescence (A, superoxide-dependent DHE; B, peroxide-dependent DCF-DA) quantified in arbitrary units in the different treatments.

ADOR induced a marked increase of O_2_·^-^ (Figure 4A–G) and peroxides (Figure 4H–N). The greater accumulation of ROS was most apparent in the apex and in the zone located 1 cm from the apex, but less marked in the elongation zone. It was also more evident in the vascular cylinder, and in the epidermis and uppermost layers (data not show).

**Figure 5:**
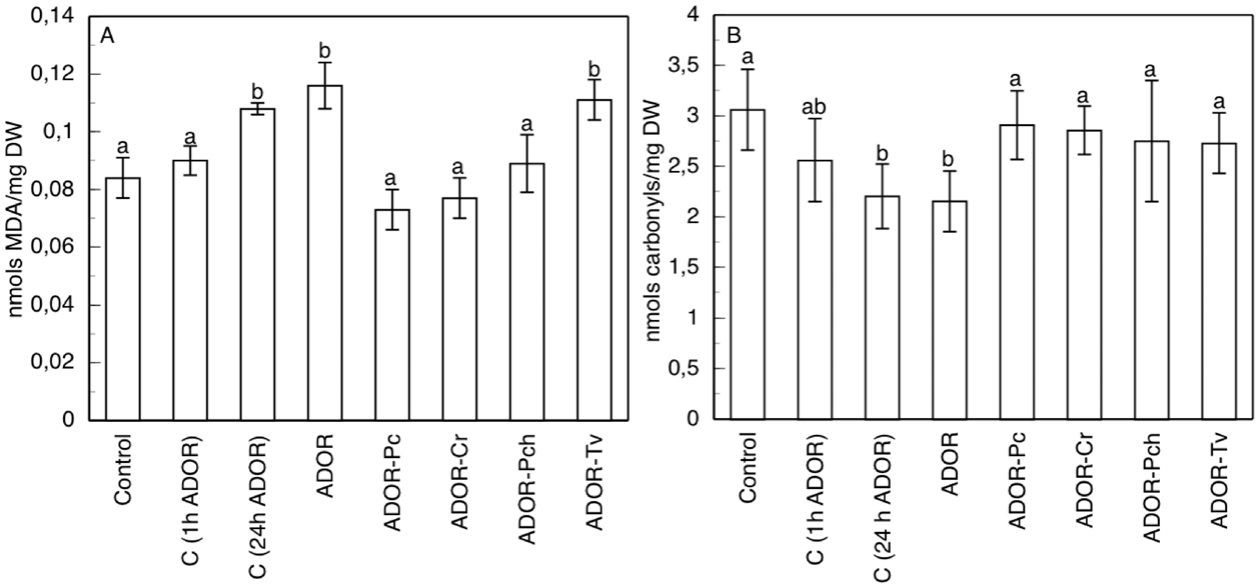
Lipid peroxidation and carbonyl concentration in sunflower roots. (A) Lipid peroxidation and (B) carbonyl concentration in sunflower roots from the different ADOR treatments. Data are means±SD of 5 independent experiments, each performed in triplicate. The means followed by the same letter are not significantly different according to Mann-Whitney U test (P≤0.05).

Both the control roots treated during 1 h or 24 h with ADOR as well as the roots germinated in ADOR, induced increases in MDA levels (Figure 5A). MDA levels in roots germinated in ADOR showed an increase of 1.4-fold the control, indicative of oxidative damage. ADOR-Pc and ADOR-Cr showed peroxidation levels below the controls.

In roots germinated and grown in ADOR (Figure 5B), there was a decrease in the levels of protein carbonylation, and a similar effect was also observed in roots germinated without ADOR and then cultured in ADOR. With the other treatments (ADOR incubated with saprobe fungi tested), however, this decline was nullified.

## Discussion

The results show that ADOR have a clear phytotoxic effect on rate and length germination and fresh weight of roots. Root length was determined from the number of dividing cells and their final cell size [57]. In this work, treated plants had higher levels of flavonoids than control plants, and shorter roots due to a decrease in meristem size. The transition zone is closer to the stem cell niche, which occurs because the meristem cells of treated roots stop dividing earlier than in the control plants, and the onset of elongation is also earlier, giving rise to shorter meristems. The accumulation of flavonoids in bean roots has also been described as inhibiting main root elongation [58], by the inhibition of the gen Cycb2–2 involved in cell division. The inhibitory effect of flavonoids was also confirmed in primary root growth in *Arabidopsis* [59]. Buer et al. [60] demonstrated that ethylene modulates flavonoid accumulation in roots of *Arabidopsis*. Treatment of wild-type Columbia seedlings with the ethylene precursor 1-aminocyclopropane carboxylic acid reduced root elongation, and the root phenotypes consistently included radial expansion. These morphological effects were not quantified, but our results suggest that the radial expansion in roots with high levels of flavonoids is a result of the greater number of cortical cell layers due to extra longitudinal divisions occurring in the outer cortex. These results show that ADOR cause direct alterations in the morphology of the meristem, resulting in reduced root length and fresh weight, and changes in the number of layers forming the cortex. These changes were partially reversed by incubation of the ADOR with saprobe fungi, recovering much of the root length and elongation zone appearing in a position above that obtained with ADOR. This shows that some components of ADOR interfere with the process of root development, and that saprobe fungi can remove, at least partially, these components. This was especially evident in the case of ADOR-Pch which like the control, had 9 layers of cortical cells, while ADOR-Cr had 10 layers and ADOR, ADOR-PC and ADOR-TV had 11 layers. These changes may also reflect a defensive response of the roots to stressors, in the form of early lignification of cell walls, thereby avoiding cell elongation similar to the obtained by Christensen et al. [30]. This would explain the reduction in total root length and FW while the DW remained essentially the same.

Under our experimental conditions, there was a decrease of PPGs in all the ADOR treatments. Flavonoids showed behaviour, with all treatments leading to increased levels. FRAP and DPPH test reveals that the roots from ADOR treatments have an impaired capacity to eliminate ROS. The results of the FRAP and DPPH tests were found to be negatively related with the flavonoid content and positively related with the PPGs. These results are in agreement with Hajdari and Mustafa's [61] who describe that flavonoids do not appear to be deeply involved in the antioxidant process of protection at the level of the roots. The effect of ADOR in decreasing the antioxidant capacity may indicate that the PPG pathway is being shifted towards producing greater lignification of the cell walls of these roots, with the resulting greater rigidity impeding cell growth. There were no significant differences in the alterations caused by incubation of ADOR with the saprobe fungi tested, all of which seemed to be capable of recovering some of the root growth. The increased flavonoid content may have acted to reduce the phytotoxicity by diminishing the amount of ROS.

Treatment of intact roots with ADOR led to rapid activation of O_2_·^-^ production. These results imply that, at short times of treatment, there is a clear defence response of the apoplast against the stress induced by ADOR, reflecting a rapid increase O_2_·^-^ production. This would be the first of the two stress induced oxidative bursts demonstrated in plants [62]. In contrast, after long treatment times, we observed a decrease in the apoplast production of O_2_·^-^. This decrease may be explained by the significant increase in SOD activity that we observed, according Yu et al. [63], except for ADOR-Pch, which presented high levels of O_2_·^-^ production and low levels of SOD activity. In the homogenates too, the observed increase in the production of O_2_·^-^ also induced increases in the SOD activity. For ADOR and ADOR-Pc homogenate the antioxidant capacity was lower and there was a greater accumulation of O_2_·^-^. The low antioxidant capacity detected in ADOR and ADOR-Pc, could be explained for the presence of some phenolic compound in the ADOR, removed by incubation with saprobe fungi [33],[35],[36], that inhibits the SOD.

ADOR was observed to induce a strong increase in the DHE-dependent fluorescence. The effect was smaller with ADOR treated with saprobe fungi. These data do not differ from those corresponding with the oxidation of epinephrine, since the fluorescence data measure both apoplastic and cytoplasmic responses, and the use of DHE in this case visualizes mainly the cytoplasmic response, showing that the stress induced by ADOR also elicits a strong response at the cytoplasm level. Treatment with ADOR induces an oxidative burst with a marked stimulation of O_2_·^-^ production and increased SOD activity. This may be due to increased SOD activity contributing to detoxifying the ROS, thus partially mitigating the effect of ADOR. Early activation of SOD in response to the oxidative stress induced by ADOR allows O_2_·^-^ to be eliminated by dismutation to H_2_O_2_, even though the total antioxidant capacity is left greatly diminished. This increase in SOD activity could explain the fluorescence imaging results of higher O_2_·^-^ levels in all the ADOR treatments. The ADOR would induce an increase in the amount of O_2_·^-^ that is formed, as evidenced by the fluorescence images, but these O_2_·^-^ would be dismutated to H_2_O_2_ by the SOD which is the only antioxidant system activated, as was observed by Sgherri et al. [64] in wheat roots subjected to Cu stress. This would explain the decrease in the amount of adrenochrome formed. Moreover, the functioning of SOD could be coupled to PM-NADPH-oxidase, to act as a catalyst for the conversion to H_2_O_2_ of the O_2_·^-^, so that extracellular SOD would be responsible for the oxidative burst [65]. Neither can one rule out a direct effect of components of the ADOR on PM-NADPH-oxidase activity.

With respect to the EC-POXs, while a rapid oxidative burst was induced in response to ADOR for DMAB-MBTH EC-POX, this was not the case for CA EC-POX. The data with the roots germinated in ADOR show declines in both of these EC-POX activities, indicating that either the induction of the response had occurred previously or such activities are being inhibited by the ADOR treatment, and hence that the behaviour is different from that observed for SOD. The DMAB-MBTH EC-POX activity in the homogenate showed smaller differences both relative to the control and between the various treatments, while the CA EC-POX activity decreased relative to the control. The sharp decline observed in EC-POX activities may also be responsible for the accumulation of H_2_O_2_, which could affect the membranes. Such effects were observed by Hossain et al. [66] who described a strong increase in SOD activity and decrease in catalase activity, resulting in greater amounts of H_2_O_2_ and oxidative damage. A similar effect is reported by Sgobba et al. [67] in TBY-2 cells exposed to heat stress. In our study, the response is consistent with the decrease in total antioxidant capacity and the consequent onset of oxidative damage caused by the accumulation of ROS in the ADOR treatments, although this was partially mitigated by incubation of the ADOR with saprobe fungi.

The fluorescence showed greater amounts of peroxide in roots grown with the ADOR treatments. This indicates that high levels of O_2_·^-^ are being produced, and that SOD activity is forming high levels of peroxide, which would also be involved in lipid peroxidation. This increase in SOD activity would explain the decline in O_2_·^-^ production, a decline that would be related to a major SOD activity rather than to any reduction in O_2_·^-^ forming capacity. The measured peroxidase activities were diminished, which also explains the accumulation of peroxides. These findings could be due to alterations at the level of either the membranes or the enzymes themselves leading to the accumulation of H_2_O_2_, as appears to be reflected in the homogenate results. Overall, there appears to be a coupling between the O_2_·^-^, SOD, and EC-POX activities, which could contribute to modulating the apoplast's accumulation of O_2_·^-^ and to greatly increase the levels of H_2_O_2_ [64].

Our results show a change in the amount of MDA in response to treatment with ADOR, indicative of oxidative damage [68],[69] with a rapid response. A similar effect was observed in the case of ADOR-Tv, but not with the other treatments. The oxidative stress induced by ADOR is similar to that observed by Zeng et al. [70] and Wang et al. [71] who report increased levels of H_2_O_2_ and MDA in response to stress caused by Hg^2+^. Similarly, this increase in MDA levels may be due to the observed increase in SOD activity and decrease in apoplast peroxidase activity, which would lead to increased levels of H_2_O_2_ [66].

No increase was observed in protein carbonylation. There was even a decrease in response to the ADOR, depending on the time of treatment. This could indicate a progressive effect on the process of root growth because, as observed by Job et al. [72] and Johansson et al. [73] in *Arabidopsis*, the pattern of protein carbonylation in plants is different from that in animals, being closely related to processes of growth, development and the plant's life cycle. Roots subjected to ADOR presented lower levels of protein carbonylation and growth than controls. But, roots obtained in ADOR incubated with saprobe fungi led to levels of protein carbonylation similar to the controls. These results would seem to indicate that oxidation of proteins in our roots is more a reflection of adaptation of the embryo and seedling to the environmental conditions during germination and culture [72] than to any response to ADOR.

Oxidative stress caused by ADOR induced a sharp increase in the proline content, confirming the findings of Tripathi and Gaur [74] and Wang et al. [71]. This increase was ADOR concentration dependent, suggesting that ADOR is in some way involved in the regulation of the proline content, although it may also reflect an increase in its synthesis and an inhibition of its oxidation [75]. There was a decrease in the total soluble amino acid content, however, which could be indicative of a modulation of the catabolism of proline in the sense that inhibition of the catabolism would lead to increased proline content. Together with the increased SOD activity, this sharp increase in proline content could explain the observed lower amount of O_2_·^-^ due to its ROS scavenging action, which might be either direct or as an agent and stabilizer of antioxidant enzymes [19]. This effect could also explain the greater decrease in O_2_·^-^ levels in roots *in vivo* than was measured in homogenates.

In sunflower seedling roots, ADOR induce changes in germination and root growth, phenolic and proline contents, and in the total antioxidant activity, inducing an oxidative stress that affects O_2_·^-^ generation, SOD, and EC-POXs activities. Treatment of the ADOR with saprobe fungi reduced its phytotoxicity by decreasing the magnitude of these effects, possibly due to remove of some phenolic compounds of the ADOR, as seen by Aranda et al. [35], [36].
